# Changes in the Epidemiology of Pneumonia in Children Younger than 14 Years Old During and After the COVID-19 Pandemic in Mexico, a National Multicenter Study

**DOI:** 10.3390/v18020270

**Published:** 2026-02-22

**Authors:** Rosa María Wong-Chew, Patricia Bautista Carbajal, Verónica Tabla-Orozco, María Del Carmen Espinosa-Sotero, Pedro Antonio Martínez-Arce, Daniel E. Noyola, María Susana Juárez-Tobías, Gerardo Martínez-Aguilar, Fabian Rojas-Larios, Izveydi Zuyino Mondragón-Salinas, Miguel Leonardo García-León

**Affiliations:** 1 Infectious Diseases Research Laboratory, Research Division, Facultad de Medicina, Universidad Nacional Autónoma de México (UNAM), Mexico City 04510, Mexico; 2 Emergency Department, Hospital Pediátrico de Coyoacán, Mexico City 04000, Mexico; 3 Department of Pediatrics, Hospital General de México Dr. Eduardo Liceaga, Mexico City 06760, Mexico; 4 Department of Pediatric Infectious Diseases, Antiguo Hospital Civil de Guadalajara Fray Antonio Alcalde, Guadalajara 44200, Mexico; 5 Center for Health Sciences and Biomedicine Research, Facultad de Medicina, Universidad Autónoma de San Luis Potosí, San Luis Potosí 78210, Mexico; dnoyola@uaslp.mx; 6 Department of Pediatrics, Hospital Regional de Alta Especialidad Dr. Ignacio Morones Prieto, San Luis Potosí 78290, Mexico; 7 Hospital Municipal del Niño de Durango, Durango 34000, Mexico; uimec@yahoo.es; 8 Facultad de Medicina y Nutrición, Universidad Juárez del Estado de Durango, Durango 34076, Mexico; 9 Hospital Regional Universitario IMSS Bienestar de Colima, Colima 28000, Mexico; frojas@ucol.mx; 10 Hospital de las Culturas, San Cristóbal de las Casas, Chiapas 29264, Mexico

**Keywords:** pneumonia, children, COVID 19 pandemic, respiratory viruses, bacteria

## Abstract

Background: In 2019, pneumonia caused 740,180 deaths in children under five years of age, representing 22% of global mortality in this age group. During the COVID-19 pandemic, public health interventions markedly reduced the circulation of most respiratory viruses other than SARS-CoV-2, leading to significant post-pandemic shifts in respiratory pathogen epidemiology. This study aimed to characterize the epidemiology, clinical features, and risk factors associated with respiratory viruses and bacteria causing pneumonia in Mexican children during the late pandemic and post pandemic periods. Methods: Children younger than 14 years with pneumonia were recruited from seven hospitals in Mexico. Demographic and clinical data were collected, and nasopharyngeal swabs were analyzed using a multiplex PCR panel detecting 19 viruses and 7 bacteria. Univariate, bivariate, and logistic regression analyses were performed (SPSS v25). Results: A total of 1715 children were included: 704 during the pandemic (2021–2023) and 1011 post-pandemic (2023–2025). Co-infections (72% vs. 65%, *p* < 0.001), virus–virus co-infections (25% vs. 11%, *p* < 0.001), and single viral infections (20% vs. 15%, *p* = 0.007) were more frequent during the pandemic. Pathogen detection was high in both periods, though negative samples increased post-pandemic (5.4% vs. 15%, *p* < 0.001). During the pandemic, the 5 most frequently detected pathogens were rhinovirus (66%), RSV A and B (38%), *Streptococcus pneumoniae* (30%), *Haemophilus influenzae* (28%), human metapneumovirus (13%). In the post-pandemic period, the 5 most frequently detected pathogens were rhinovirus (52%), *Haemophilus influenzae* (36%), *Streptococcus pneumoniae* (35%), RSV A and B (28%), metapneumovirus (11%). Rhinovirus and RSV predominated during the pandemic, whereas *Haemophilus influenzae*, *Streptococcus pneumoniae*, parainfluenza viruses, *Bordetella pertussis*, and *Mycoplasma pneumoniae* significantly increased post-pandemic. Conclusions: Pediatric pneumonia epidemiology shifted from a predominantly viral profile during the pandemic to increased bacterial detections and virus–bacteria co-infections post-pandemic, alongside re-emergence of typical RSV and influenza seasonality. Higher mean age and rhinovirus as the most frequent pathogen persist after the pandemic. Sustained molecular surveillance and reinforced vaccination programs remain essential in the post-pandemic era.

## 1. Introduction

Lower respiratory tract infections (LRTIs) remain the deadliest transmissible diseases worldwide and rank as the fourth leading cause of death across all age groups. Although mortality has declined over time, an estimated 2.6 million deaths were attributed to LRTIs in 2019, representing approximately 460,000 fewer deaths compared with the year 2000 [1]. Globally, 740,180 children under five years of age died from pneumonia in 2019, accounting for 22% of all deaths in this age group, with the highest mortality burden observed in Southeast Asia and sub-Saharan Africa [2]. In Mexico, according to the National Institute of Geography and Statistics (INEGI), influenza and pneumonia ranked as the third leading cause of death among children younger than five years in 2018 [3].

Pneumonia can be caused by viruses, bacteria, or fungi. Among viral pathogens, respiratory syncytial virus (RSV) and human rhinovirus are the most frequently detected; among bacterial agents, *Streptococcus pneumoniae* and *Haemophilus influenzae* type b predominate; and among fungal pathogens, *Pneumocystis jirovecii* is primarily observed in immunocompromised individuals, particularly patients with HIV infection [2].

The widespread implementation of multiplex reverse transcription polymerase chain reaction (RT-PCR) assays during the 2010s enabled more comprehensive etiological characterization of pediatric pneumonia. Consequently, several large epidemiological studies describing respiratory pathogens in children hospitalized with pneumonia were published during this decade. In the United States, Jain et al. reported a multicenter study conducted in three hospitals that included 2222 children younger than 18 years of age between 2010 and 2012. The median age was 2 years (interquartile range, 1–6 years), and at least one pathogen was detected in 81% of cases. Viruses were identified in 66% of patients, bacterial pathogens alone in 8%, and viral–bacterial coinfections in 7%. The highest incidence occurred in children younger than two years of age. The most frequently detected pathogens were RSV (28%), human rhinovirus (27%), human metapneumovirus (13%), adenovirus (11%), *Mycoplasma pneumoniae* (8%), parainfluenza viruses (7%), influenza viruses (7%), coronaviruses (5%), *S. pneumoniae* (4%), *Staphylococcus aureus* (1%), and *Streptococcus pyogenes* (1%). RSV (37% vs. 8%), adenovirus (15% vs. 3%), and human metapneumovirus (15% vs. 8%) were more frequently detected in children younger than five years compared with older children, whereas *M. pneumoniae* was more common among children older than five years (19% vs. 3%). A total of 497 children (21%) required admission to the intensive care unit, and three patients (1%) died [4].

Our group previously described, for the first time in Mexico, the viral etiology of pneumonia in 1404 children younger than five years of age treated at 11 hospitals across the country between 2010 and 2013. Respiratory viruses were detected in 81% of cases. In descending order of frequency, the pathogens identified were RSV A and B (23.7%), human rhinovirus/enterovirus (17%), human metapneumovirus (5.7%), parainfluenza viruses types 1–4 (5.5%), influenza viruses (3.6%), adenovirus (2.2%), human coronaviruses NL63, OC43, 229E, and HKU1 (2.2%), and human bocavirus (0.4%). Viral coinfections involving up to five viruses were observed in 22% of cases, with RSV and rhinovirus being the most frequent combination. The mean age of patients was 14 months, and 41% of cases occurred during winter, followed by 30% during autumn. Bacterial pathogens were not evaluated in this study [5].

At the onset of the COVID-19 pandemic in 2020, public health and social measures—including mask use, hand hygiene, social distancing, and enhanced epidemiological surveillance—were widely implemented. These interventions contributed not only to the containment of SARS-CoV-2 but also to a marked reduction in the circulation of other respiratory viruses [6,7]. By mid-2021, however, the circulation of several respiratory viruses resumed. Their epidemiological behavior was atypical, with outbreaks occurring outside the usual seasonal patterns, particularly during summer and autumn, and with higher incidence rates than those observed in pre-pandemic periods in multiple regions worldwide, including the United States, Australia, and Japan [7]. Similar post-pandemic alterations in the epidemiology of pediatric respiratory infections have been widely reported in Europe. Several countries documented delayed, off-season, or intensified outbreaks of respiratory syncytial virus (RSV) and influenza following the relaxation of non-pharmaceutical interventions. In France, an interseasonal resurgence of RSV was observed in children shortly after the lifting of public health measures, with epidemic timing and age distribution differing from pre-pandemic patterns [8]. Comparable phenomena were described in other European settings, including the United Kingdom and multinational surveillance networks, where marked reductions in invasive respiratory bacterial diseases during the pandemic were followed by heterogeneous rebounds after mitigation measures were eased [9].

In this context, the aim of the present study was to describe changes in pathogen detection patterns and epidemiological trends of respiratory viruses and bacteria in children younger than 14 years with clinically and/or radiologically diagnosed pneumonia during the second half of the COVID-19 pandemic and the post-pandemic period, across seven hospitals in Mexico.

## 2. Materials and Methods

### 2.1. Study Population and Study Design

A prospective cross-sectional, multicenter observational study was conducted. Patients were recruited between April 2021 and November 2025 from seven hospitals in Mexico: Hospital Pediátrico de Coyoacán (Mexico City); Hospital General de México “Dr. Eduardo Liceaga” (Mexico City); Hospital Regional de Alta Especialidad “Dr. Ignacio Morones Prieto” (San Luis Potosí); Antiguo Hospital Civil de Guadalajara “Fray Antonio Alcalde” (Guadalajara, Jalisco); Hospital Municipal del Niño de Durango (Durango); Hospital Regional Universitario IMSS-Bienestar de Colima (Colima); and Hospital de las Culturas (San Cristóbal de las Casas, Chiapas).

Children younger than 14 years of age with a clinical and/or radiological diagnosis of pneumonia were eligible for inclusion. Clinical pneumonia was defined by the presence of respiratory distress, cough, tachypnea, xiphoid or costal retractions, cyanosis, with or without fever, within the first week of symptom onset. Radiological pneumonia was defined by chest X-ray findings showing pulmonary infiltrates classified as macronodular, micronodular, lobar, interstitial, multifocal, pleural effusion, or mixed patterns. Risk factors assessed at admission included: Daycare attendance, Immunocompromised, Breastfeeding, Household tobacco exposure, Influenza vaccination (last year), Pneumococcal vaccination, Household income and type of co-infection.

A standardized electronic case report form was developed for the study. Demographic and clinical data were collected locally at each participating institution and subsequently centralized and processed at the Faculty of Medicine, National Autonomous University of Mexico (UNAM).

### 2.2. Ethical Statement

The study protocol was approved by the ethics and research committees of all participating institutions (Facultad de Medicina UNAM FM/DI/105/2020; Antiguo Hospital Civil de Guadalajara “Fray Antonio Alcalde” 052/20; Hospital Municipal del Niño de Durango 001/2021; Hospital Regional de Alta Especialidad “Dr. Ignacio Morones Prieto” 37-21; Hospital General de México “Dr. Eduardo Liceaga” DI/22/505/05/42; Hospital Pediátrico de Coyoacán 101-011-025-21; Hospital Regional Universitario de Colima CI 2021/02/CR/PED/130, Hospital de las Culturas, San Cristobal de las Casas, Chiapas). Written informed consent was obtained from the parents or legal guardians of all participants prior to patient enrollment.

### 2.3. Study Procedures and Sample Collection

Parents or legal guardians of eligible patients presenting to the emergency department or requiring hospitalization were invited to allow their children to participate in the study. After written informed consent was obtained and clinical data were recorded, a nasopharyngeal swab sample was collected from each participant.

Samples were placed in 5 mL sterile plastic tubes containing viral transport medium and stored at −70 °C at each participating center. Specimens were transported every 4–6 months to the Infectious Diseases Research Laboratory at the Faculty of Medicine, UNAM, where they were stored at −70 °C until further processing.

Testing practices remained consistent throughout the study period. All participating centers applied the same clinical indications for respiratory pathogen testing, used nasopharyngeal swabs collected at hospital admission, and followed standardized laboratory workflows for multiplex RT-PCR testing. The same standardized methods for sampling procedures, assay platforms, or interpretation criteria were performed over time. The primary difference between periods was the inclusion of one additional participating hospital (Chiapas) during the post-pandemic phase.

### 2.4. Multiplex RT-PCR for the Detection of Respiratory Pathogens

The samples were processed at the Infectious Diseases Research Laboratory, Faculty of Medicine, UNAM. Seegene’s SRARMag96X4 Universal Cartridge kit (Seegene, Seoul, Republic of Korea) was used for the automatic RNA extraction; 50–60 μL of the sample were added to 96-well plates into the Bio-Rad’s C1000 Thermo Cycler to perform the real time multiplex PCR.

The Allplex™ Respiratory Infection Full Panel (Seegene, Seoul, Republic of Korea) was performed on a Bio-Rad CFX96 Dx real-time PCR platform and interpreted using Seegene Viewer software version 3.34.001. This multiplex real-time RT-PCR assay is based on MuDT™ technology and provides individual cycle threshold (Ct) values for each detected target. Result interpretation (positive/negative) was performed automatically by Seegene Viewer software version 3.34.001 according to the manufacturer’s validated Instructions for Use, which integrate amplification curve characteristics, internal controls, and assay-specific analytical thresholds. Although Ct values were recorded for all detected pathogens, no fixed Ct cut-off was applied by the investigators to define positivity or etiological relevance, as the manufacturer does not establish clinically validated Ct thresholds for this purpose. The assay detects 19 respiratory viruses (Influenza A, H1, H1N1, H3, and B, parainfluenza 1, 2, 3, and 4, adenovirus, bocavirus, respiratory syncytial virus A, B, metapneumovirus A/B, coronavirus NL63, 229E, OC43, rhinovirus, enterovirus), and 7 bacteria (*M. pneumoniae*, *B. pertussis*, *B. parapertussis*, *C. pneumoniae*, *H. influenzae*, *S. pneumoniae*, and *L. pneumophila*).

In addition to the Allplex™ Respiratory Infection Full Panel, detection of SARS-CoV-2 was performed using the Allplex™ SARS-CoV-2/FluA/FluB/RSV Assay (Seegene, Seoul, Republic of Korea). Both assays are multiplex real-time RT-PCR tests and were performed on nasopharyngeal swab samples using standardized laboratory procedures. Results from the SARS-CoV-2 assay were integrated at the patient level with those obtained from the respiratory panel for descriptive and epidemiological analyses.

### 2.5. Definition of Severe Pneumonia

Severe pneumonia was defined according to the World Health Organization (WHO) criteria [10]. Children were classified as having severe pneumonia if they presented with cough or difficulty breathing accompanied by at least one general danger sign (inability to drink or breastfeed, persistent vomiting, convulsions, lethargy or unconsciousness), severe respiratory distress, central cyanosis, or hypoxemia requiring oxygen supplementation. Cases requiring admission to the intensive care unit were also classified as severe pneumonia, in accordance with WHO guidelines.

### 2.6. Statistical Analysis

Statistical analyses were performed using the Statistical Package for the Social Sciences (IBM^®^ SPSS^®^ Statistics version 25). Univariate and bivariate analyses were conducted. Categorical variables were compared using the chi-square test or Fisher’s exact test, as appropriate, and continuous variables were analyzed using Student’s *t*-test. A *p* value < 0.05 was considered statistically significant.

Multivariable logistic regression analysis was performed to identify risk factors associated with severe pneumonia. Incidence density for each viral and bacterial pathogen was calculated as the number of detected cases per 10 newly diagnosed pneumonia cases per month at each participating center. This metric was used as a relative frequency index to compare the temporal distribution of pathogens across centers and study periods, rather than as a population-based incidence rate [11].

## 3. Results

The sanitary emergency due to the COVID-19 pandemic in Mexico was officially declared on 31 March 2020, following the implementation of non-pharmacological interventions, including social distancing, beginning on 23 March 2020. The end of the sanitary emergency was declared by the World Health Organization on 5 May 2023, and by Mexican health authorities on 9 May 2023. According to records from the Mexican Ministry of Health, the circulation of most respiratory viruses markedly declined starting in March 2020, with a gradual re-emergence beginning in July 2021.

This study compares the clinical characteristics, epidemiological features, and risk factors of pediatric pneumonia cases from July 2021 to April 2023, corresponding to the second half of the pandemic period, and from May 2023 to November 2025, corresponding to the post-pandemic period, across seven hospitals in Mexico.

### 3.1. Demographic Characteristics

A total of 1715 pediatric patients were included in the analysis: 704 during the pandemic period (July 2021–April 2023) and 1011 during the post-pandemic period (May 2023–November 2025). The mean age (mean ± SE) was 1.95 ± 0.10 years during the pandemic and 2.16 ± 0.10 years in the post-pandemic period (*p* = 0.60). Mean weight was 12.02 ± 0.35 kg versus 13.16 ± 0.37 kg (*p* = 0.31), and mean height was 0.81 ± 0.01 m versus 0.81 ± 0.09 m (*p* = 0.44), respectively. Male patients accounted for 45% versus 47% and females for 55% versus 53% during and after the pandemic, respectively (*p* = 0.35). There were no differences for immunocompromise between the 2 periods.

Regarding hospital distribution, 31% of patients were from Hospital Pediátrico de Coyoacán (Mexico City), 22% from Hospital Regional de Alta Especialidad “Dr. Ignacio Morones Prieto” (San Luis Potosí), 19% from Antiguo Hospital Civil de Guadalajara “Fray Antonio Alcalde”, 17.03% from Hospital General de México “Dr. Eduardo Liceaga” (Mexico City), 6.6% from Hospital de las Culturas (San Cristóbal de las Casas, Chiapas), 4% from Hospital Regional Universitario IMSS-Bienestar de Colima, and 0.82% from Hospital Municipal del Niño de Durango (Table 1).

### 3.2. Clinical Characteristics

Most patients were managed in general wards during both periods (86% vs. 88%), followed by outpatient management (11% vs. 10%) and intensive care unit admission (3% vs. 2.5%), with no statistically significant differences between periods (*p* = 0.57). Severe pneumonia, as defined by the World Health Organization classification, occurred in 191 of 704 patients (27.1%) during the pandemic period and in 232 of 897 patients (25.9%) during the post-pandemic period. The difference between periods was not statistically significant (*p* = 0.568).

The mean respiratory rate at admission during the pandemic and post-pandemic periods was 38.0 ± 0.52 versus 40.26 ± 0.69 breaths per minute (*p* = 0.85). Mean heart rate was 130.98 ± 0.89 bpm versus 131.4 ± 1.0 bpm (*p* = 0.41), and mean temperature was 36.9 ± 0.03 °C versus 37.21 ± 0.03 °C (*p* < 0.001), respectively.

Cough was reported in 89% of cases during the pandemic versus 79% after the pandemic (*p* = 0.02). Respiratory distress was present in 83% versus 74% (*p* = 0.36). Xiphoid retractions were observed in 36% versus 31% (*p* = 0.69), intercostal retractions in 60% versus 58% (*p* = 0.01), nasal flaring in 32% versus 32% (*p* = 0.11), thoracoabdominal dissociation in 38% versus 39% (*p* = 0.73), dysphonia in 15% versus 15.0% (*p* = 0.23), and chest pain in 9.8% versus 12.0% (*p* = 0.02). Radiographic patterns during and after the pandemic included interstitial infiltrates (47% vs. 43%), lobar consolidation (13% vs. 5.7%), micronodular patterns (7.9% vs. 13%), macronodular patterns (1.1% vs. 1.88%), multifocal involvement (1.4% vs. 2.6%), pleural effusion (0.71% vs. 0.79%), and mixed patterns (13% vs. 5.6%) (Table 2).

### 3.3. Pathogens Detected by Multiplex PCR

During the pandemic period, 1515 pathogens were detected in 704 nasopharyngeal samples, including 161 single infections (23%) and 505 co-infections (72%). In the post-pandemic period, 2058 pathogens were identified in 1011 samples, with 203 single infections (20%) and 655 co-infections (60%). Negative samples accounted for 5.4% during the pandemic and increased to 15% in the post-pandemic period (*p* < 0.001).

Overall, co-infections (72% vs. 65%, *p* < 0.001), virus–virus co-infections (25% vs. 11%, *p* < 0.001), and single viral infections (20% vs. 15%, *p* = 0.007) were more frequent during the pandemic. In contrast, virus–bacteria co-infections (46% vs. 52%, *p* = 0.016), bacteria–bacteria co-infections (0.57% vs. 2%, *p* = 0.012), and single bacterial detections (2.7% vs. 4.9%, *p* = 0.027) pandemic vs. post-pandemic, respectively, were more frequent in the post-pandemic period (Table 3).

During the pandemic, the most frequently detected pathogens were rhinovirus (66%), RSV A and B (38%), *Streptococcus pneumoniae* (30%), *Haemophilus influenzae* (28%), human metapneumovirus (13%), SARS-CoV-2 (10%), bocavirus (9.1%), parainfluenza viruses types 1, 3, and 4 (5.3%), influenza A and B (2.4%), adenovirus (2.73%), human coronaviruses OC43, 229E, and NL63 (2.94%), and *Mycoplasma pneumoniae* (0.14%). Thirty-eight samples (5.4%) were negative.

In the post-pandemic period, the most frequently detected pathogens were rhinovirus (52%), *Haemophilus influenzae* (36%), *Streptococcus pneumoniae* (35%), RSV A and B (27.9%), human metapneumovirus (11%), influenza A and B (9.2%), parainfluenza viruses (7.8%), SARS-CoV-2 (4.3%), *Bordetella pertussis/parapertussis* (4%), bocavirus (3.6%), adenovirus (2.9%), *Mycoplasma pneumoniae* (2.7%), human coronaviruses OC43, 229E, and NL63 (1.9%), *Chlamydia pneumoniae* (0.3%), and *Legionella pneumophila* (0.09%). A total of 153 samples (15%) were negative (Table 3).

When comparing both periods, a statistically significant decrease in the frequency of rhinovirus, RSV B, SARS-CoV-2, bocavirus, and enterovirus was observed in the post-pandemic period, whereas parainfluenza viruses, influenza B, *Haemophilus influenzae*, *Bordetella pertussis*, and *Mycoplasma pneumoniae* showed a significant increase (Table 3).

### 3.4. Pathogens Detected by Age Group

**Figure 1:**
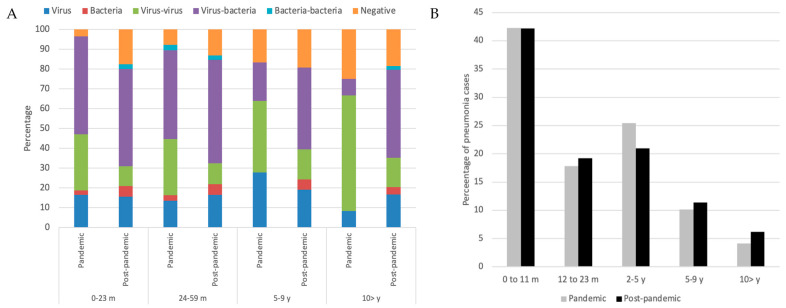
Viral and bacterial pathogens according to age groups detected during and after the pandemic in children with pneumonia in 7 hospitals in Mexico. (**A**) Percentage of virus and bacteria in single or co-infections according to age and period (during and after the pandemic). (**B**) Percentage of pneumonia cases according to age groups during and after the pandemic.

During the pandemic, viral–viral and viral–bacterial co-infections were observed across all age groups, with an overall reduction after the pandemic. The highest number of detected pathogens occurred in children younger than five years, particularly in the 0–23-month age group, followed by the 2–4-year group. In this youngest group, co-infections—both viral–viral and viral–bacterial—were particularly frequent. Bacterial detections were most common in the 2–4-year age group during the pandemic, and viral–bacterial co-infections increased after the pandemic across all age groups. In children aged 10–14 years, a higher proportion of viral–viral detections was observed during the pandemic. Overall, more than 75% of most pathogens were detected as part of co-infections during the pandemic period (Figure 1A).

The age distribution of pneumonia cases showed that 60% versus 61% (*p* = 0.86) occurred in the 0–23-month age group, 25% versus 21% (*p* = 0.06) in the 2–4-year group, 10% versus 11% (*p* = 0.43) in the 5–9-year group, and 4.1% versus 6.2% (*p* = 0.09) in the 10–14-year group during and after the pandemic, respectively (Figure 1B).

**Figure 2:**
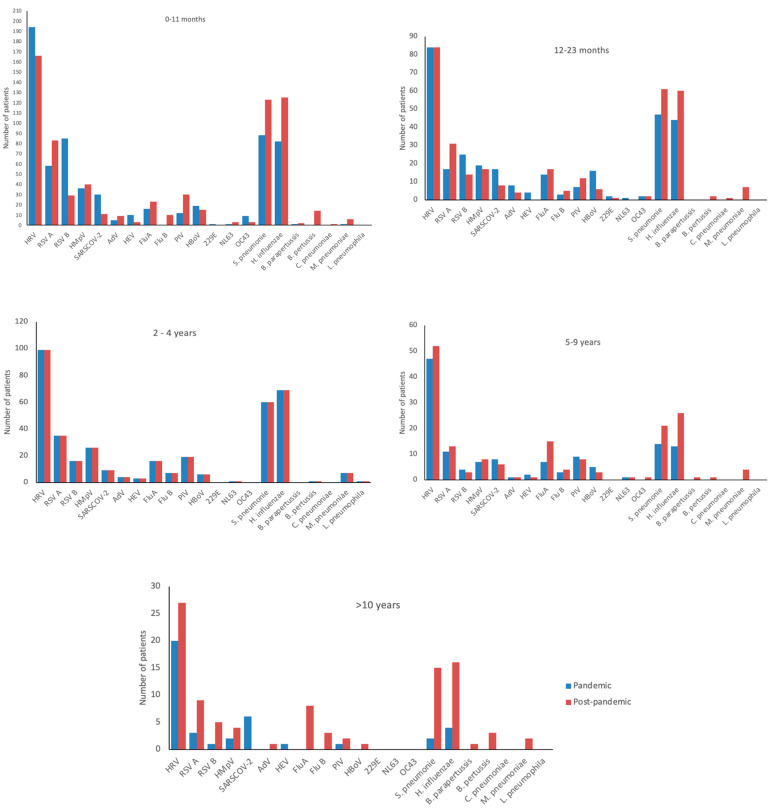
Pathogens detected according to different age groups during and after the pandemic.

Across all age groups and both periods, rhinovirus was the most frequently detected virus. Detailed pathogen distributions by age group are shown in Figure 1 and Figure 2. When restricting the analysis to viral infections, rhinovirus remained the predominant pathogen in all age groups, followed by RSV and human metapneumovirus in children younger than five years. SARS-CoV-2 ranked as the fourth most frequent virus overall during the pandemic but was the second most frequent in the 10–14-year age group; after the pandemic, its frequency markedly declined, with no detections in children older than 10 years. Influenza virus detections increased after the pandemic in all age groups, although cases were already observed during the pandemic, particularly in children aged 5–9 years. In all age groups, viral–viral co-infections decreased after the pandemic, whereas viral–bacterial co-infections increased (Table 4).

Regarding disease severity, 27% of cases during the pandemic met criteria for severe pneumonia compared with 23% in the post-pandemic period (*p* = 0.044). By age group, severe pneumonia occurred in 31% versus 26% of children aged 0–23 months, 22% versus 20% in those aged 2–4 years, 21% versus 11% in those aged 5–9 years, and 17% versus 6% in those aged 10–14 years during and after the pandemic, respectively.

### 3.5. Seasonality

**Figure 3:**
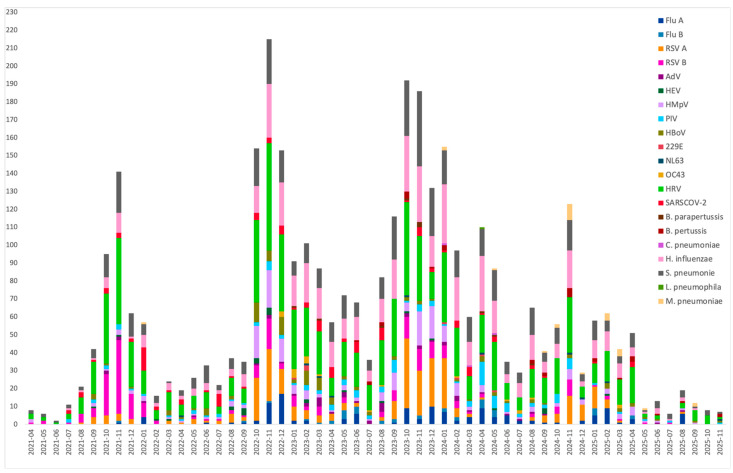
Seasonality of viral and bacterial pathogens detected in children with pneumonia from April 2021 to October 2024 in 7 hospitals in Mexico.

Seasonal analysis showed that pneumonia cases peaked predominantly during fall and winter from 2021 to 2024. RSV and influenza viruses displayed clear seasonal patterns, with circulation beginning in autumn and peaking in winter. In contrast, rhinovirus, *Streptococcus pneumoniae*, and *Haemophilus influenzae* were detected throughout the year. SARS-CoV-2 was present year-round, with a pronounced peak in January 2022 corresponding to the predominance of the Omicron variant in Mexico. Bocavirus was detected year-round, with a marked increase between October 2022 and March 2023 (Figure 3).

### 3.6. Incidence of Viral and Bacterial Pathogens in Children with Pneumonia

**Figure 4:**
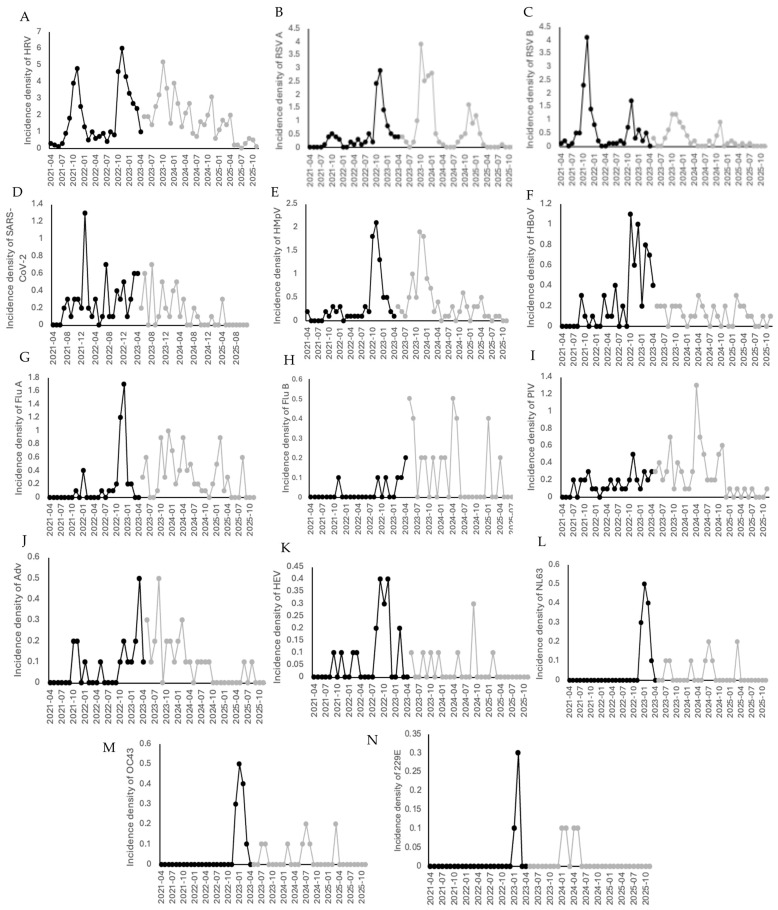
Incidence density of viral infections before and after the pandemic in children in 7 hospitals in Mexico. (**A**) Rhinovirus, (**B**) RSV A, (**C**) RSV B, (**D**) SARS-CoV-2, (**E**) metapneumovirus, (**F**) bocavirus, (**G**) influenza A, (**H**) influenza B, (**I**) parainfluenza, (**J**) adenovirus, (**K**) enterovirus, (**L**) coronavirus NL63, (**M**) coronavirus OC43, (**N**) coronavirus 229E. Black lines correspond to the pandemic period, gray lines correspond to the post-pandemic period.

Incidence density was calculated as the number of detected pathogens per 10 new pneumonia cases per month. Rhinovirus showed the highest incidence during the pandemic, with two winter peaks in 2021 and 2022, and continued to circulate year-round in the post-pandemic period. RSV exhibited a seasonal autumn–winter pattern; RSV B predominated in 2021, whereas RSV A became the predominant subtype from 2022 onward. SARS-CoV-2, human metapneumovirus, and bocavirus showed a decrease in incidence during the post-pandemic period. Influenza A peaked in 2022 and subsequently exhibited a seasonal pattern, while parainfluenza viruses increased in frequency after the pandemic. Human coronaviruses OC43, NL63, and 229E were detected at low frequency during the pandemic and showed increased circulation from 2023 onward. Adenovirus and enterovirus were detected in both periods (Figure 4).

**Figure 5:**
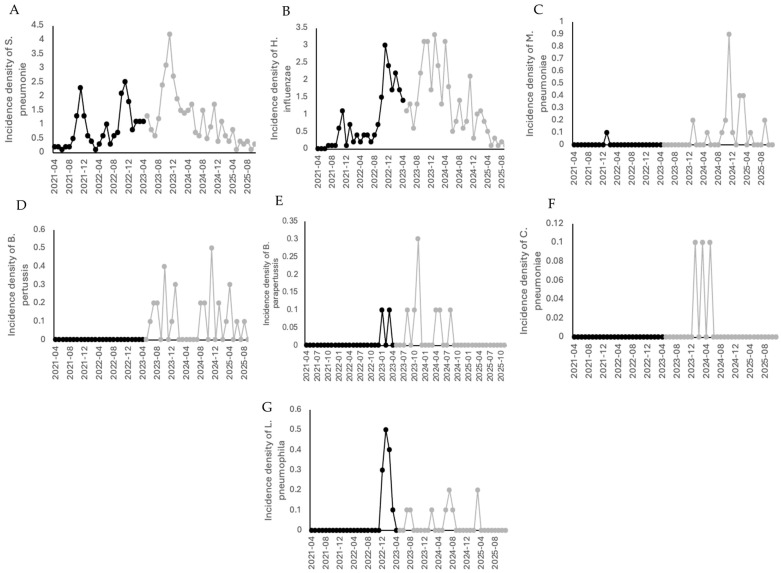
Incidence density of bacterial infections before and after the pandemic in children in 7 hospitals in Mexico. (**A**) *Streptococcus pneumoniae*, (**B**) *Haemophilus influenzae*, (**C**) *Mycoplasma pneumoniae*, (**D**) *Bordetella pertussis*, (**E**) *Bordetella parapertussis*, (**F**) *Chlamydia pneumoniae*, (**G**) *Legionella pneumophila*. Black lines correspond to the pandemic period; gray lines correspond to the post-pandemic period.

*Streptococcus pneumoniae* and *Haemophilus influenzae* were detected throughout both periods, with *H. influenzae* showing an increased incidence after the pandemic. *Mycoplasma pneumoniae*, *Bordetella pertussis*, *Bordetella parapertussis*, *Chlamydia pneumoniae*, and *Legionella pneumophila* were not detected during the pandemic but increased in frequency during the post-pandemic period (Figure 5).

### 3.7. Risk Factors

Reported risk factors during and after the pandemic included prior SARS-CoV-2 infection (5% vs. 4%), daycare attendance (13% vs. 13%), immunocompromised status (6% vs. 5%), absence of breastfeeding (22% vs. 21%), mixed breastfeeding (19% vs. 18%), household tobacco exposure (41% vs. 36%), lack of influenza vaccination (69% vs. 69%), and lack of pneumococcal vaccination (31% vs. 38%), respectively (Table 5).

Multivariable logistic regression analysis identified pneumococcal vaccination during the post-pandemic period as a protective factor against severe pneumonia (OR 0.67; 95% CI 0.46–0.97; *p* = 0.035). In contrast, high household income was associated with an increased risk of severe pneumonia (OR 4.17; 95% CI 1.39–12.55; *p* = 0.011). No other evaluated risk factors were significantly associated with disease severity (Table 5).

## 4. Discussion

In this multicenter study, we describe changes in the epidemiology, clinical presentation, and etiology of pediatric pneumonia during the second half of the COVID-19 pandemic and the post-pandemic period in Mexico. Although demographic characteristics and age distribution were similar across periods, we observed differences in disease severity, pathogen distribution, coinfection patterns, and seasonality.

Severe pneumonia, defined according to World Health Organization criteria, occurred at similar proportions during the pandemic and post-pandemic periods. These findings suggest that, despite substantial shifts in respiratory pathogen circulation and coinfection patterns, overall clinical severity remained stable across study periods within participating centers. While COVID-19-related non-pharmaceutical interventions (NPIs) significantly altered the epidemiology of respiratory infections at the population level, such changes were not accompanied by measurable differences in the proportion of severe pneumonia in this cohort [12,13]. In addition, indirect effects of the pandemic on pediatric healthcare use and severe disease presentations have been documented, supporting the plausibility that pandemic-era conditions could be associated with differences in pneumonia severity profiles [14]. Another finding was that the mean age of presentation during and after the pandemic persist at 2 years, whereas before the pandemic the mean age was 14 months [14].

Clinical manifestations differed modestly between periods. During the pandemic, cough and respiratory distress were reported more frequently, whereas post-pandemic cases showed higher body temperatures and a higher frequency of chest pain. These findings may reflect changes in the etiological composition of pneumonia across periods, particularly the increased detection of bacterial pathogens and virus–bacteria coinfections after the pandemic. At a broader scale, population-level reductions and subsequent rebounds in respiratory pathogen circulation following NPI implementation have been described for multiple pathogens [9,12].

A key finding of this study is the shift in pathogen detection patterns between periods. During the pandemic, viral infections—particularly virus–virus coinfections and single viral detections—were more frequent, whereas the post-pandemic period showed a higher proportion of bacterial detections, including virus–bacteria and bacteria–bacteria coinfections. Similar shifts in respiratory and invasive bacterial disease epidemiology have been reported during the pandemic, likely reflecting changes in transmission opportunities under mitigation measures rather than pathogen-specific effects [9].

Rhinovirus was the most frequently detected pathogen across all age groups in both periods. This finding is consistent with reports indicating that rhinovirus transmission in children persisted despite social restrictions, suggesting relative resistance of rhinovirus circulation to certain NPIs [15]. The highest frequency of rhinovirus persisted after the pandemic, in contrast to pre pandemic periods where RSV was the second most frequent [5]. RSV circulation recovered over time and exhibited seasonal patterns, with changes consistent with delayed and atypical epidemics reported following the implementation and relaxation of public health measures in different countries [8,16]. These international observations support the plausibility of the temporal shifts observed in our incidence and seasonality analyses.

The post-pandemic increase in RSV, influenza viruses, parainfluenza viruses, and atypical bacterial pathogens such as *Mycoplasma pneumoniae* and *Bordetella* spp. may reflect increased population susceptibility following prolonged periods of reduced exposure. This phenomenon has been described as an “immunity debt” and has been proposed as a contributing factor to atypical resurgences after the relaxation of NPIs, although its magnitude and mechanisms remain under discussion [13]. For *M. pneumoniae*, global surveillance data demonstrated a marked reduction in detections during the pandemic compared with pre-pandemic years, supporting the interpretation that transmission was suppressed during periods of strict mitigation [17].

Age-stratified analyses showed that children younger than five years accounted for most pneumonia cases in both periods, with the highest burden in those aged 0–23 months. This group also exhibited the highest proportion of co-infections. Systematic reviews and meta-analyses have shown that respiratory viral coinfections are common in pediatric populations, although their association with disease severity varies across studies [18,19]. Our findings suggest that coinfection patterns differed by period, with fewer virus–virus and more virus–bacteria coinfections after the pandemic, a shift that may have implications for clinical interpretation and management.

Regarding seasonality, RSV and influenza showed recovery of typical autumn–winter patterns, whereas rhinovirus and the bacterial targets included in the panel were detected throughout the year. SARS-CoV-2 remained detectable year-round, with a pronounced peak corresponding to the Omicron wave in early 2022, consistent with WHO epidemiological reporting [20]. Of note, during the post-pandemic period, SARS-CoV-2 was not detected in individuals older than 10 years, suggesting prior immunity and reduced disease severity in older children following the pandemic. Bocavirus was detected throughout the study period and showed a transient increase in late 2022 and early 2023. Given that prolonged shedding and frequent detection of bocavirus in young children have been documented, its detection should be interpreted cautiously when attributing causality in pneumonia cases [21].

Incidence density analyses were consistent with these observations, showing sustained rhinovirus circulation across periods and increased detection of several bacterial pathogens after the pandemic. The absence of *Mycoplasma pneumoniae* detections during the pandemic and its emergence afterward in our cohort is directionally consistent with global surveillance findings reporting reduced circulation during periods of strict NPIs [17].

The interpretation of multiplex RT-PCR results in pediatric pneumonia requires careful consideration. The use of nasopharyngeal samples limits etiological inference, as detection of a respiratory pathogen does not necessarily indicate causality. Several bacterial targets included in the panel, particularly *Streptococcus pneumoniae* and *Haemophilus influenzae*, are well-recognized colonizers of the upper respiratory tract in healthy children, with nasopharyngeal carriage rates reported to reach 30–40% depending on age, vaccination status, and setting [22,23]. Similarly, respiratory viruses can be detected in a substantial proportion of asymptomatic children when highly sensitive molecular methods are used, complicating the attribution of causality in lower respiratory tract infections [24,25]. Therefore, molecular detection in the upper respiratory tract should be interpreted as an association rather than definitive evidence of causation.

The high frequency of viral–viral and viral–bacterial coinfections observed in this study further supports this interpretation. Co-infections detected by molecular assays do not necessarily imply simultaneous etiological roles of all identified pathogens, but rather reflect complex interactions between host susceptibility, pathogen circulation, and environmental exposure. Previous systematic reviews and meta-analyses have shown that respiratory viral coinfections are common in children, while their association with disease severity remains inconsistent across studies [18,19]. In this context, coinfections may serve as epidemiological markers of exposure and altered transmission dynamics during and after the COVID-19 pandemic rather than direct drivers of pneumonia severity.

Some pathogens detected in this study are classically associated with specific respiratory syndromes, such as RSV with bronchiolitis or *Bordetella pertussis* with whooping cough. However, these agents have also been reported in children hospitalized with clinical and radiological diagnoses of pneumonia, particularly in young infants and severe cases [4,26]. The overlap between respiratory syndromes in pediatric patients highlights the clinical complexity of lower respiratory tract infections and supports a cautious, syndrome-based interpretation of molecular findings rather than strict pathogen-specific attribution.

With respect to risk factors for severe pneumonia, pneumococcal vaccination was associated with a lower risk of severity in the post-pandemic period. This association is consistent with evidence showing reduced rates of community-acquired pneumonia in children following widespread implementation of pneumococcal conjugate vaccines [27]. In contrast, higher household income was associated with increased severity in our model. This finding should be interpreted cautiously, as it may reflect healthcare-seeking behavior, referral patterns to tertiary hospitals, or residual confounding rather than a direct causal relationship. Interpretation of socioeconomic variables in hospital-based studies requires consideration of broader social determinants and access-related pathways [28].

Some limitations of the study include its hospital-based design which limits extrapolation to community-managed pneumonia. The use of nasopharyngeal samples limits etiologic inference for both bacterial and viral respiratory detections, as bacterial colonization and viral asymptomatic carriage or prolonged shedding cannot be distinguished from lower respiratory tract infection. Although immuno-compromised status was recorded, other comorbidities associated with pneumonia severity—such as chronic cardiac or pulmonary diseases, prematurity, hematologic disorders, metabolic conditions, and obesity—were not systematically collected across all sites. Therefore, residual confounding related to unmeasured predisposing conditions cannot be excluded. Finally, changes in healthcare access and testing practices over time may have influenced detection patterns, although standardized procedures were applied across centers. However, because the national epidemiological surveillance system primarily captures severe respiratory cases and only approximately 10% of non-severe cases, and initially test influenza and SARS-CoV-2 and only in 10% of the negatives the rest of viruses, this study provides nationally relevant information on the pathogens detected in children with pneumonia who are not necessarily severely ill or who are managed in outpatient settings, as comprehensive testing for all pathogens allow estimation of their relative proportions.

In conclusion, our findings suggest that pediatric pneumonia epidemiology in Mexico shifted from a predominantly viral pattern during the pandemic toward increased bacterial detections and virus–bacteria coinfections in the post-pandemic period, alongside recovery of typical seasonality for RSV and influenza. Higher mean age and rhinovirus as the most frequent pathogen persist after the pandemic. These observations underscore the importance of sustained molecular surveillance and vaccination strategies in the post-pandemic era.

## Figures and Tables

**Table 1:** Demographic characteristics of children with pneumonia during and after the pandemic in Mexico.

Variable	Pandemic	Post-Pandemic	*p*
	*n* = 704	*n* = 1011	
Age (years), x ± SE	1.95± 0.10	2.16 ± 0.10	0.60
Weight (kg), x ± SE	12.02 ± 0.35	13.16 ± 0.37	0.31
Height (m), x ± SE	0.81 ± 0.01	0.81 ± 0.09	0.44
Male, *n* (%)	317 (45)	479 (47)	0.35
Female, *n* (%)	387 (55)	532 (53)	
Immunocompromise, *n* (%)	45 (6.4)	52 (5.1)	0.76
**Hospital**			**Total**
HP Coyoacán, *n* (%)	181 (26)	348 (34)	529 (31)
HRAE de SLP, *n* (%)	213 (30)	162 (16)	375 (22)
AHC Guadalajara, *n* (%)	156 (22)	165 (16)	321 (19)
HGMDEL, *n* (%)	94 (13)	198 (19)	292 (173)
HCSCC Chiapas, *n* (%)	0 (0)	114 (11)	114 (6.6)
HRUIB Colima, *n* (%)	46 (6.5)	24 (2.3)	70 (4)
HMI Durango, *n* (%)	14 (2)	0 (0)	14 (0.82)

HP Coyoacán—Hospital Pediátrico de Coyoacán, HRAE de SLP—Hospital Regional de Alta especialidad Ignacio Morones Prieto of San Luis Potosí, AHC Guadalajara—Antiguo Hospital Civil de Guadalajara Fray Antonio Alcalde, HGMDEL—Hospital General de Mexico Dr. Eduardo Liceaga HCSCC Chiapas—Hospital de las Culturas de San Cristobal de las Casas, Chiapas., HRUIB Colima—Hospital Regional Universitario IMSS Bienestar de Colima, HMI Durango—Hospital Municipal del Niño de Durango.

**Table 2:** Clinical characteristics of children with pneumonia during and after the pandemic.

Variable	Pandemic		Post-Pandemic		*p*
	*n* = 704	*n* evaluated	*n* = 1011	*n* evaluated	
Ambulatory, *n* (%)	79 (11)	79/704	99 (9.8)	99/1011	0.540
Hospitalization, *n* (%)	604 (86)	604/704	886 (88)	886/1011	
ICU, *n* (%)	21 (3)	21/704	26 (2.6)	26/1011	
Severe pneumonia, *n* (%)	191 (27)	191/704	232 (23)	232/897 *	0.568
Temperature (°C), x ± SE	36.9 ± 0.03		37.21 ± 0.03		**<0.001**
Respiratory rate (breaths/min) x ± SE	38.43 ± 0.52		40.26 ± 0.69		0.850
Heart rate, (beats/min) x ± SE	130.98 ± 0.89		131.4 ± 1.0		0.410
Respiratory distress, *n* (%)	584 (83)	584/699	746 (74)	746/874	0.360
Xiphoid retractions, *n* (%)	256 (36)	256/695	311 (31)	311/870	0.690
Intercostal retractions, *n* (%)	419 (59)	419/696	581 (57)	581/871	**0.010**
Nasal flaring, *n* (%)	227 (32)	227/692	321 (32)	321/872	0.110
Thoracoabdominal dissociation, *n* (%)	264 (37)	264/692	342 (39)	342/874	0.730
Dysphonia, *n* (%)	104 (15)	104/692	152 (15)	152/873	0.230
Chest pain, *n* (%)	69 (9.8)	69/690	121 (12)	121/871	**0.020**
Cough, *n* (%)	627 (89)	627/699	807 (80)	807/867	**0.021**
Interstitial, *n* (%)	331 (47)	331/592	434 (43)	434/737	**<0.001**
Lobar, *n* (%)	91 (13)	91/592	58 (5.7)	58/737	
Macronodular, *n* (%)	8 (1.1)	8/592	19 (1.9)	19/737	
Micronodular, *n* (%)	56 (7.9)	56/592	135 (13.3)	135/737	
Multiple foci, *n* (%)	10 (1.4)	10/592	26 (2.6)	26/737	
Mixed, *n* (%)	91 (13)	91/592	57 (5.6)	57/737	
Pleural effusion, *n* (%)	5 (0.71)	5/592	8 (0.79)	8/737	

In bold are the statistically significant differences. Chi-square tests were performed *Per protocol*, using available cases for each variable due to missing data. * Because the Chiapas center was incorporated exclusively during the post-pandemic period and reported no severe pneumonia cases, a sensitivity analysis excluding this center was conducted to assess the robustness of severity comparisons between periods.

**Table 3:** Pathogens detected in nasopharyngeal samples of children with pneumonia during and after the pandemic in 7 hospitals in Mexico.

	Pandemic Total Detections * n = 1515			Post-Pandemic Total Detections * n = 2058			*p*
	Patients *n* = 704 *n* (%)	Single Viral Agent *n* = 142 *n* (%)	Single Bacterial Agent *n* = 19 *n* (%)	Patients *n* = 1011 *n* (%)	Single Viral Agent *n* = 153 *n* (%)	Single Bacterial Agent *n* = 50 *n* (%)	
Human rhinovirus	463 (66)	84 (59.15)		523 (52)	71 (46.41)		**<0.001**
RSV A	121 (17)	9 (6.34)		200 (20)	38 (24.84)		0.196
RSV B	148 (21)	16 (11.27)		80 (7.9)	7 (4.58)		**<0.001**
Human metapneumovirus	88 (13)	12 (8.45)		116 (11)	16 (10.46)		0.568
SARS-CoV-2	71 (10)	9 (6.34)		44 (4.3)	4 (2.61)		**<0.001**
Human bocavirus	64 (9.1)	4 (2.82)		37 (3.6	1 (0.65)		**<0.001**
Parainfluenza 1, 3, 4	37 (5.3)	6 (4.23)		79 (7.8)	6 (3.92)		**0.047**
Influenza A *	29 (4.1)	0 (0)		59 (5.8)	0 (0)		0.140
Influenza B	7 (1)	0 (0)		34 (3.4)	4 (2.61)		**0.002**
Adenovirus	19 (2.7)	0 (0)		29 (2.9)	1 (0.65)		0.951
Human Enterovirus	19 (2.7)	1 (0.70)		9 (0.89)	0 (0)		**0.006**
Coronavirus 229E	4 (0.57)	0 (0)		4 (0.39)	0 (0)		0.876
Coronavirus NL63	4 (0.57)	0 (0)		6 (0.59)	0 (0)		1.0
Coronavirus OC43	13 (1.8)	1 (0.70)		9 (0.89)	0 (0)		0.130
*Streptococcus pneumoniae*	214 (30)		8 (42.11)	352 (35)		26 (52)	0.062
*Haemophilus influenzae*	196 (28)		11 (57.89)	370 (36)		20 (40)	**<0.001**
*Bordetella pertussis*	0 (0)		0 (0)	32 (3.2)		1 (2)	**<0.001**
*Bordetella parapertussis*	2 (0.28)		0 (0)	8 (0.79)		0 (0)	0.30
*Chlamydia pneumoniae*	0 (0)		0 (0)	3 (0.30)		0 (0)	0.390
*Mycoplasma pneumoniae*	1 (0.14)		(0)	27 (2.7)		3 (6)	**<0.001**
*Legionella pneumophila*	0 (0)		(0)	1 (0.09)		0 (0)	1.0
Negative	38 (5.4)			153 (15)			**<0.001**
Co-infections	505 (72)			655 (65)			**<0.001**
Virus-virus co-infections	177 (25)			108 (11)			**<0.001**
Virus-bacteria co-infections	324 (46)			526 (52)			**0.016**
Bacteria-bacteria co-infections	4 (0.57)			21 (2)			**0.012**
Single infections	161 (23)			203 (20)			0.183
Single viral infection	142 (20)			153 (15)			**0.007**
Single bacterial infection	19 (2.7)			50 (4.9)			**0.027**

* Total detections refer to the total number of detected pathogens, counting each pathogen identified in a given sample separately, including multiple detections within the same patient. In bold are statistically significant differences.

**Table 4:** Frequency of viral pathogens according to age group during and after the pandemic.

	0–23 Months		2–5 Years		5–9 Years		10–14 Years	
	Pandemic *n* = 424 *n* (%)	Post-Pandemic *n* = 621 *n* (%)	Pandemic *n* = 179, *n* (%)	Post-Pandemic *n* = 212 *n* (%)	Pandemic *n* = 72 *n* (%)	Post-Pandemic *n* = 115 *n* (%)	Pandemic *n* = 29 *n* (%)	Post-Pandemic *n* = 63 *n* (%)
HRV	278 (66)	250 (40)	115 (64)	99 (47)	47 (65)	52 (45)	20 (69)	27 (43)
RSV A/B	185 (44)	157 (25)	63 (35)	51 (24)	15 (21)	16 (14)	4 (14)	14 (22)
MpV	55 (13)	57 (9.2)	24 (13)	26 (12)	7 (9.7)	8 (7)	2 (6.9)	4 (6.3)
SARS-CoV-2	47 (11)	19 (3) *	9 (5)	9 (4.2)	8 (11)	6 (5.2)	6 (21)	0 (0) *
Influenza A/B	33 (7.8)	55 (8.8) *	7 (3.9)	23 (11) *	10 (14)	19 (17)	0 (0)	11 (17)
HBoV	35 (7.8)	21 (3.4)	24 (13)	6 (2.8) *	5 (6.9)	3 (2.6)	0 (0)	1 (1.6)
PIV	19 (4.5)	42 (6.7) *	9 (5)	19 (9) *	9 (13)	8 (7)	1 (3)	2 (3.2)
Coronavirus	16 (3.7)	9 (1.4)	4 (2.2)	1 (0.47)	1 (1.4)	1 (0.87)	0 (0)	0 (0)
Adenovirus	13 (3.8)	13 (2)	5 (2.8)	4 (1.9)	1 (1.4)	1 (0.87)	0 (0)	1 (1.6)
Enterovirus	4 (0.94)	1 (0.16)	2 (1.1)	3 (1.4)	2 (2.8)	1 (0.87)	1 (3.4)	0 (0)
Total co-infections	322 (76)	327 (53) *	133 (74)	118 (56) *	36 (50)	56 (49)	11 (38)	32 (51) *
Virus-virus co-infections	112 (26)	53 (8.5) *	43 (24)	19 (9) *	14 (19)	15 (13)	7 (24)	8 (13)
Virus-bacteria co-infections	210 (49)	261 (42)	86 (48)	95 (45)	22 (31)	41 (36)	4 (14)	24 (38)
Severe pneumonia	131 (31)	171 (26)	40 (22)	43 (20)	15 (21)	13 (11)	5 (17)	4 (6.3)

* Statistically significant differences between the pandemic and post pandemic proportions. HRV—human rhinovirus, RSV—Respiratory syncytial virus, MpV—Metapneumovirus, SARS-CoV-2—Severe acute respiratory syndrome Coronavirus 2, HBoV—Human bocavirus, PIV—parainfluenza viruses. The *n* is the number of patients of each age group and each period.

**Table 5:** Risk factors associated with severe pneumonia in children younger than 14 years old in 7 hospitals in Mexico. In bold are statistically significant differences.

Variable		Pandemic (*n* = 704)				Post-Pandemic (*n* = 1011)			
		*n* (%)	OR	95% CI	*p*	*n* (%)	OR	95% CI	*p*
Previous SARS-CoV-2 infection		38 (5)	0.64	0.25–1.62	0.341	46 (4)	0.44	0.18–1.07	0.071
Daycare attendance		94 (13)	0.73	0.42–1.28	0.278	141 (14)	0.79	0.49–1.26	0.316
Immunocompromised		45 (6)	1.49	0.75–2.97	0.254	52 (5)	1.08	0.57–2.05	0.806
Breastfeeding	Absent vs. Exclusive	158 (22)	0.74	0.46–1.17	0.192	213 (21)	0.91	0.61–1.34	0.62
	Mixed vs. Exclusive	134 (19)	0.9	0.55–1.45	0.661	184 (18)	0.75	0.49–1.14	0.175
Household tobacco exposure		292 (41)	0.82	0.57–1.19	0.294	364 (36)	1.09	0.78–1.5	0.62
Influenza vaccination (last year)		222 (31)	0.93	0.62–1.4	0.733	313 (31)	0.94	0.65–1.34	0.718
Pneumococcal vaccination		484 (68)	0.85	0.56–1.29	0.436	632 (62)	0.67	0.46–0.97	**0.035**
Household income	High vs. Middle	4 (0.6)	2.44	0.31–19.47	0.399	18 (2)	4.17	1.39–12.55	**0.011**
	Low vs. Middle	576 (81)	0.89	0.54–1.44	0.627	610 (60)	1.46	0.98–2.15	0.061
Type of co-infection	Viral–bacterial vs. None	324 (46)	1.28	0.82–2.0	0.284	526 (52)	1.06	0.74–1.5	0.762
	Viral–viral vs. None	177 (25)	1.46	0.89–2.4	0.132	108 (10)	1.05	0.61–1.81	0.852

## Data Availability

De-identified individual-level data for both cohorts and the accompanying data dictionary are available from the corresponding author upon reasonable request and subject to institutional and ethics approval due to patient privacy considerations.
